# Reverse transcription-quantitative PCR (RT-qPCR) without the need for prior removal of DNA

**DOI:** 10.1038/s41598-023-38383-4

**Published:** 2023-07-15

**Authors:** Damir Đermić, Sven Ljubić, Maja Matulić, Alfredo Procino, Maria Chiara Feliciello, Đurđica Ugarković, Isidoro Feliciello

**Affiliations:** 1 Division of Molecular Biology, Ruder Boskovic Institute, 10000 Zagreb, Croatia; 2 Division of Molecular Biology, Department of Biology, Faculty of Science, University of Zagreb, 10000 Zagreb, Croatia; 3 Department of Statistical Science, Alma Mater Studiorum, University of Bologna, 40126 Bologna, Italy; 4 Department of Clinical Medicine and Surgery, University of Naples Federico II, 80135 Naples, Italy

**Keywords:** Genetics, Molecular biology

## Abstract

The procedure illustrated in this paper represents a new method for transcriptome analysis by PCR (Polymerase Chain Reaction), which circumvents the need for elimination of potential DNA contamination. Compared to the existing methodologies, our method is more precise, simpler and more reproducible because it preserves the RNA’s integrity, does not require materials and/or reagents that are used for elimination of DNA and it also reduces the number of samples that should be set up as negative controls. This novel procedure involves the use of a specifically modified primer during reverse transcription step, which contains mismatched bases, thus producing cDNA molecules that differ from genomic DNA. By using the same modified primer in PCR amplification, only cDNA template is amplified since genomic DNA template is partially heterologous to the primer. In this way, amplification by PCR is unaffected by any potential DNA contamination since it is specific only for the cDNA template. Furthermore, it accurately reflects the initial RNA concentration of the sample, which is prone to changes due to various physical or enzymatic treatments commonly used by the current methodologies for DNA elimination. The method is particularly suitable for quantification of highly repetitive DNA transcripts, such as satellite DNA.

## Introduction

The qualitative and/or quantitative analysis of transcripts by PCR amplification is a method of choice in both molecular biology research and medical diagnostics^1^. It is widely used to detect and quantify many different types of transcripts such as messenger RNA, ribosomal RNA, non-coding RNA etc. The most common applications include gene expression analysis and precise identification of a particular microorganism.

In order to be properly analyzed, the purified RNA is subjected to a preliminary and fundamental step of reverse transcription, through which the RNA molecules are converted into cDNA (complementary DNA) by the reverse transcriptase enzyme. Indeed, the RNA itself cannot be directly amplified during the subsequent PCR steps and must necessarily be converted into cDNA^1^. The main problem of most currently used protocols lies in the often-present DNA contamination, which is impossible to chemically differentiate on a structural level from the cDNA by the polymerase enzyme during PCR amplification, thus causing false positive results^2–7^. In order to overcome this limitation, all current protocols include a couple of DNA elimination steps, both during the purification of RNA and subsequent reverse transcription step. In both cases the DNA would be eliminated, either with the aid of specific mechanical filters (silica-based columns) or through enzymatic digestion by a specific enzyme, such as DNase I (Deoxyribonuclease I)^8^. However, these treatments are not 100% effective for removal of DNA, which often remains as DNA contamination^2,3^. This is particularly the case with highly repetitive DNA which constitutes a substantial part of eukaryotic genome and is often transcribed at low levels. Furthermore, it should be emphasized that any procedure implemented to reduce the concentration of DNA in the sample certainly also causes the reduction of initial concentration of the RNA itself, a molecule which is by its very nature unstable and easily degradable.

Here we report a novel method for transcriptome analysis by PCR, wherein cDNA and DNA are differentiated and thus contamination by the latter is excluded, hence producing more precise, reliable and reproducible results.

## Materials and methods

### Construction of primers

Forward, reverse and modified primers used to test the new protocol are illustrated in Table 1. The modified specific primer differs with respect to the unmodified forward and reverse primers in just four base alterations (point mutations) distributed alternatively with unchanged nucleotides and starting from the 3’ end of the primer (marked in bold in Table 1).

**Table 1:** List of primers used in transcription analyses.

	Forward	Reverse	PSM
*ssb*	GTTGTGCTGTTCGGCAAACT	GCGATCCTGACCGGATTGAT	GCGATCCTGACCG**C**A**A**T**C**A**A**
*sulA*	CCTGAACCCATTCCCGACTC	GCCGGGCTTATCAGTGAAGT	CCTGAACCCATTC**G**C**C**A**G**T**G**
*recA*	AGGGCGTCACAGATTTCCAG	TTCCGGTAAAACCACGCTGA	AGGGCGTCACAGA**A**T**A**C**G**A**C**
TCAST1	CCATAAGCGAGTTATAGAGTTGG	CTTTAGTGACTTTTATGTCTTCTCC	CCATAAGCGAGTTATA**C**A**C**T**A**G**C**
RPS18	CGAAGAGGTCGAGAAAATCG	CGTGGTCTTGGTGTGTTGAC	CGTGGTCTTGGTG**A**G**A**T**C**A**G**
ASAT	CACTCTTTTTGTAGAATCTGC	AATGCACACATCACAAAGAAG	AATGCACACATCAC**T**A**T**G**T**A**C**
GUSB	GAAAATACGTGGTTGGAGAGCTCATT	CCGAGTGAAGATCCCCTTTTTA	CCGAGTGAAGATCCC**G**T**A**T**A**T**T**

Changes in PSM relative to reverse or forward primer are shown in bold.

We also tested primers that were modified in other ways (from two to six transitions/transversions at 3’ end, with and without aforementioned alternation) but the best, most consistent results were obtained with 4 alternating mismatches at 3’ end for 20–26 bp long primers. Of course, for longer primers more mutations can be included, but in our experience, four mismatches proved to be a necessary and sufficient number of modifications in order to achieve the expected results. In our case, the modified primers altered the complementarity between the primer and the target, while preserving the specificity and thermodynamics of primers themselves. The list of all designed primers is shown in Table 1.

### RNA isolation and reverse transcription for prokaryotic gene analysis

An *Escherichia coli* strain (AB1157) used to test *ssb, sulA* and *recA* gene expression contained a multicopy plasmid, pID2, for increased expression of *ssb* gene^9^. *ssb* (coding for an essential, conserved SSB protein, which regulates DNA metabolism), *sulA* (under SOS control, coding for SulA inhibitor of cell division) and *recA* (coding for a conserved bacterial recombinase, RecA). The bacteria were grown in LB medium, with aeration, at 37 °C until they reached OD_600_ ~ 0.4 and afterwards RNA was isolated from them.

RNA was purified by RNeasy Plus Mini kit (Qiagen) which, according to the manufacturer’s instructions, includes a genomic DNA elimination step by solid phase column extraction. Samples treated in such a way are indicated as + DNase I and those untreated for genomic DNA elimination are denoted as – DNase I.

Approximately 1 μg of RNA, quantified by Quant-IT RNA using a Qubit fluorometer (Invitrogen, Waltham, MA, USA), was reverse transcribed using the PrimeScript RT reagent kit without gDNA Eraser (+ RT/– DNase I) or with gDNA Eraser (+ RT/ + DNase I) (Takara, Dalian, China) using a 0.2 µM mix of specifically modified or random hexamer primers (from the kit) for *ssb*, *sulA* and *recA* expression analysis, for both the new and current method. In summary, the samples designated + DNase I had their DNA eliminated by both silica-based column during RNA isolation and by gDNA eraser (DNase I treatment) during reverse transcription step. For all samples, negative controls without reverse transcriptase enzyme (-RT) were prepared.

### Nanoplate based digital PCR (dPCR) for absolute quantification of bacterial gene expression

Nanoplate based dPCR procedure was performed using the QIAcuity 2-plex instrument (Qiagen, Hilden, Germany). The dPCR reaction mixture was assembled using QIAcuity 3X Eva Green PCR Master Mix, 10X primer mix (2 µM), RNase-free water and a fixed concentration of cDNA template in a final volume of 15 µL per sample. After accurate vortexing, 12 µL of the prepared mixture was transferred onto the 24-well 8.5 K nanoplate and sealed with nanoplate rubber seal. For quality control of QIAcuity dPCR, the assay was replicated with different amounts of cDNA template input (24, 12, and 6 ng), quantified by Quant-IT ssDNA using a Qubit fluorometer (Invitrogen, Waltham, MA, USA). The sequences of primers for transcript detection of *ssb,* s*ulA,* and *recA* genes are indicated in Table 1.

The results for all samples were obtained using 24 ng template input except for *ssb* gene expression analysis in the current method, which was performed using 60 × diluted target sample with respect to samples used for the new method due to the presence of multicopy plasmid expressing *ssb* gene (Fig. 2A).

The 8.5 K nanoplate gives rise to 8500 single partitions in which the template is distributed randomly. The QIAcuity carries out fully automated sample processing, including all necessary steps for plate priming, sealing of partitions, thermocycling and image analysis. The amplification cycling protocol consisted of 95 °C for 2 min for enzyme activation step and the following 40 cycles of 15 s. at 95 °C for denaturation, 15 s. at 60 °C for annealing, and 15 s. at 72 °C for extension, concluding with the final step at 40 °C for 5 min. Fluorescent light is emitted by positive partitions that contain a target molecule, as opposed to those without it, the negative partitions. Data were analyzed using the QIAcuity Suite Software V1.1.3 (Qiagen) and the results were expressed as copies of cDNA/µl based on Poisson distribution analyses. The partitions produced by the machine resemble Poisson process since the targets end up in different partitions independently and with a fixed rate. The Poisson distribution gives probabilities for positive integer random events. The key parameter of this distribution is the expectation value for these events, which means it is the mean probability for a proportion of a counting process or the counting process per se for the dPCR analysis. Furthermore, the QIAcuity has embedded software that can quantify and produce reliable statistics. In our case the statistical measure we considered was the Poisson confidence interval at a 95% level that, when plotted (error bar), shows whether or not the events differ with 95% confidence.

### RNA isolation and reverse transcription for satellite DNA analysis

Alpha satellite (ASAT) RNA was isolated from cervical cancer human cell line HeLa (obtained from ATCC (USA) using the RNeasy Plus Mini kit (Qiagen). Cells were cultivated in DMEM supplemented with 10% Fetal Bovine Serum (both from Sigma, MA USA), in humidified atmosphere of 5% CO_2_ and on 37 °C.) TCAST1 satellite RNA was isolated from *Tribolium castaneum* adult beetles using the RNeasy Plus Mini kit (Qiagen) which includes a genomic DNA elimination step by solid phase column extraction. Approximately 1 μg of RNA, quantified by Quant-IT RNA using a Qubit fluorometer (Invitrogen, Waltham, MA, USA), was reverse transcribed using the PrimeScript RT reagent kit with gDNA Eraser (Takara, Dalian, China) in 10 μL reaction solution, using specifically modified primers for ASAT and TCAST1 expression analysis. For all samples, negative controls without reverse transcriptase enzyme were also prepared.

### Quantitative real-time PCR (qPCR) for satellite DNA expression analysis

qPCR analysis was performed according to the previously published protocol^10,11^. Primers for the expression analysis of human alpha satellite DNA were constructed according to the alpha satellite consensus sequence^12^ and TCAST1 primers according to its own satellite consensus sequence^13^. *Glucuronidase β (GUSB*-Gene ID: 2990) and ribosomal protein S18 (RPS18) were used as endogenous controls for normalisation in human and *Tribolium* samples, respectively, and were stably expressed without any significant variation among samples. The following thermal cycling conditions were used: 50 °C 2 min; 95 °C 7 min; 95 °C 15 s; 60 °C 1 min for 40 cycles followed by dissociation stage: 95 °C for 15 s; 60 °C for 1 min; 95 °C for 15 s; and 60 °C for 15 s. Amplification specificity was confirmed by dissociation curve analysis and the specificity of amplified products was tested on agarose gel. Control without template (NTC) was included in each run. Post-run data were analysed using LinRegPCR software v.11.1. which enables calculation of the starting concentration of amplicon in the sample (“N0 value”). N0 value is expressed in arbitrary fluorescence units and is calculated by considering PCR efficiency and baseline fluorescence. “N0 value” determined for each technical replicate was averaged and the averaged “N0 values” were divided by the “N0 values” of the endogenous control (Figs. 5B and 6B). In this paper we decided also to show the graphical representation of Delta Rn vs Cycle raw data amplification plots (Figs. 5A and 6A, Suppl. Fig. 1).

## Results and discussion

### Description of the proposed method

**Figure 1:**
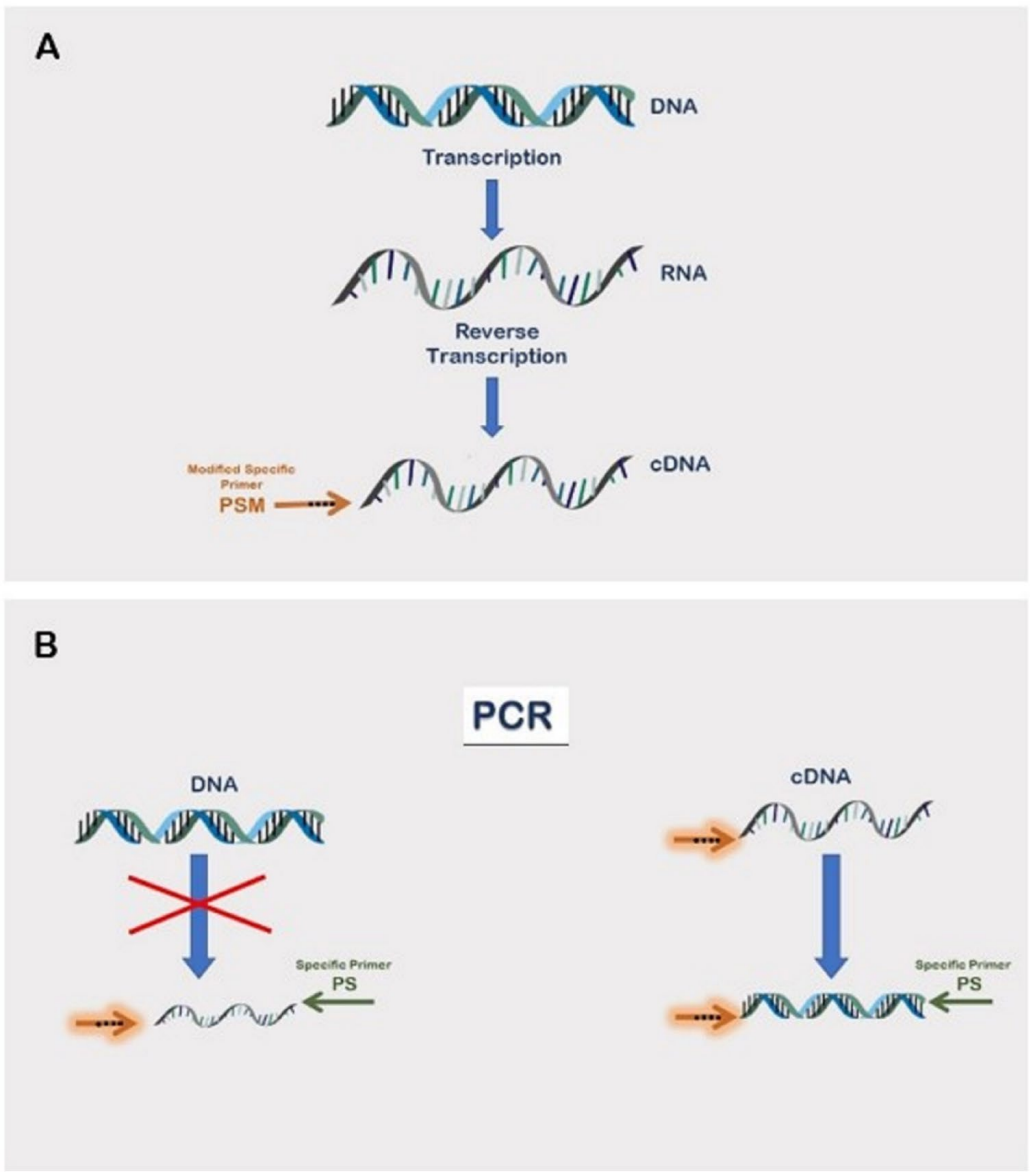
Schematic illustration of the new method. (**A**) Basic model of nucleic acid metabolism from DNA to cDNA. Integration of modified specific primer into cDNA by means of reverse transcription makes it a permanent part of the sequence. (**B**) Amplification of target sequence by means of polymerase chain reaction. cDNA converted by modified specific primer is properly amplified at certain discriminating temperature, while genomic DNA targets are successfully avoided.

Our proposed method, schematically depicted in Fig. 1, takes advantage of using a modified primer (Modified Specific Primer, PSM) during the reverse transcription step of the protocol. Such a primer is specific for the RNA molecules to be quantified and its nucleotide sequence is designed to lack a perfect homology to the retro-transcribed template DNA. Generally, it is enough to add few mismatches with respect to the original sequence, preferably located in the close proximity to the 3'-OH terminal region. These modifications make the primer partially complementary to the target sequence but still able to hybridize at the temperatures of 37–42 °C used during the reverse transcription step. Nevertheless, the PSM will dissociate from the partially homologous genomic DNA sequence during the PCR step, once the operating temperature reaches around 60 °C. The aim of using such conveniently modified specific primer is to achieve amplification specifically from cDNA template while successfully avoiding genomic DNA targets. The correct number of modifications to be applied, their effectiveness and proper discriminating temperatures should be experimentally tested for each and every transcript to be analyzed, by selecting those parameters that show negative and positive amplification tendencies towards DNA and cDNA targets, respectively. This optimization phase represents a preliminary step of our method that enables the setup of negative and positive controls and, advantageously, has to be carried out only once, since it always remains valid for a specific amplicon and can be applied to a varying number of replicates under different experimental conditions. Indeed, in current protocols the negative control (NC: – RT, without reverse transcriptase) should ideally be prepared for each new sample to be tested, even though the target is the same, due to the random effectiveness of DNase I treatment. Using a PSM we are able to generate cDNA slightly different from its genomic DNA counterpart, due to the nucleotide mismatches present in the sequence.

During the phase following reverse transcription (Fig. 1B), the amplification of cDNA by PCR takes place using the same modified primer (PSM) from the previous step in addition to the unmodified specific primers (SP) starting from the opposite direction. Consequently, the resulting amplicon is a copy of the cDNA and not the DNA, due to the specifically selective annealing temperatures usually ranging from 55 °C to 62 °C. Therefore, with this procedure, there is no need to eliminate the co-purified DNA from the RNA sample since it is no longer a competing target and will not affect the final result of the assay. Indeed, in certain experimental conditions it could be useful and advantageous to have both DNA and RNA present together in the same sample if, for example, the results need to be normalized with respect to the gene copy number variation.

### Validation of the method

**Figure 2:**
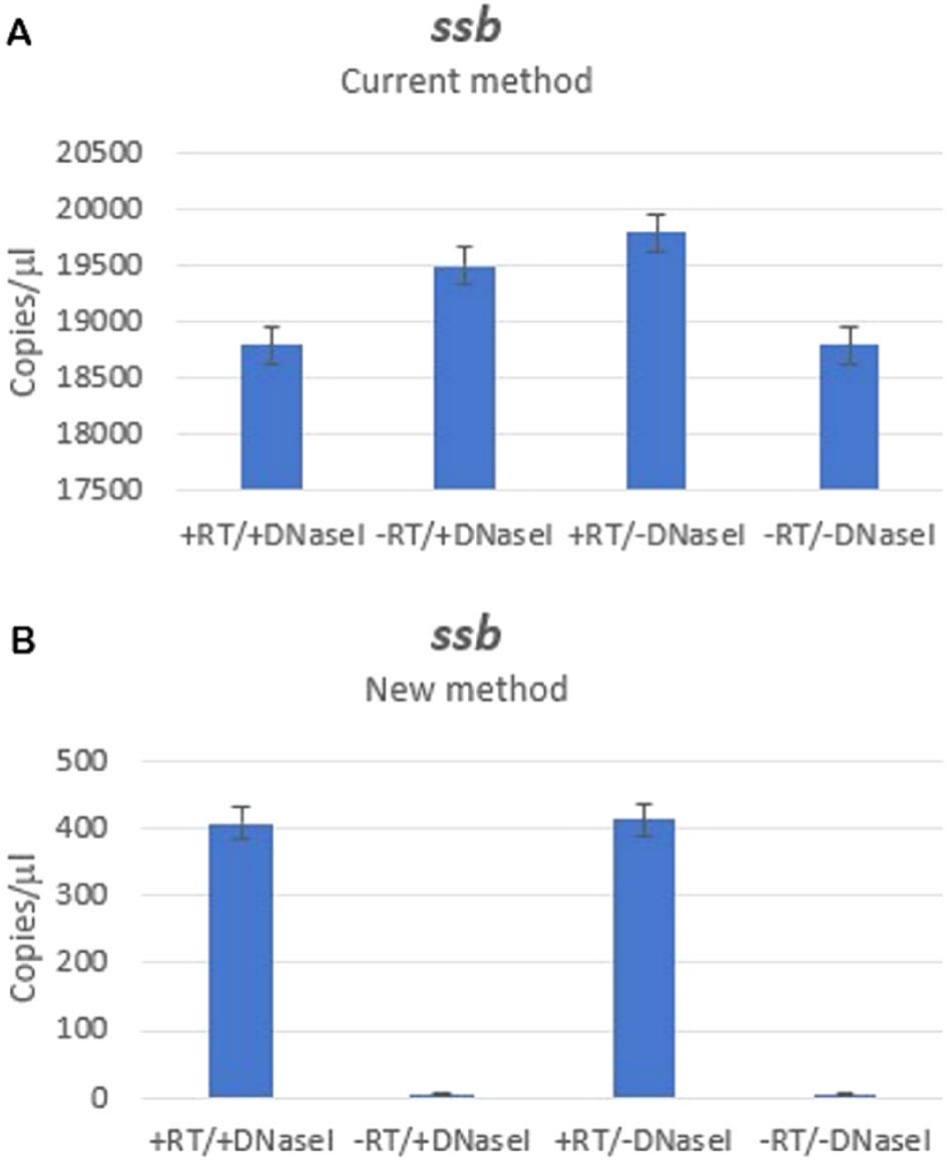
Transcription of *ssb* gene in exponentially growing *E. coli* cells harbouring *ssb* overexpression plasmid pID2 obtained by dPCR using current (**A**) and new method (**B**). Columns represent number of copies/µl and the plotted error bar shows whether or not the events differ with 95% Poisson confidence interval.

**Figure 3:**
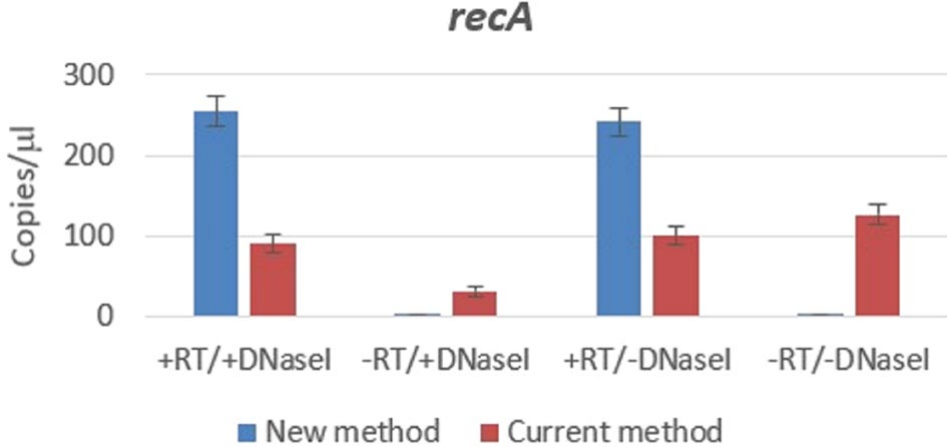
Transcription of *recA* gene in exponentially growing *E. coli* cells obtained by dPCR using current and new method. Columns represent number of copies/µl and the plotted error bar shows whether or not the events differ with 95% Poisson confidence interval.

**Figure 4:**
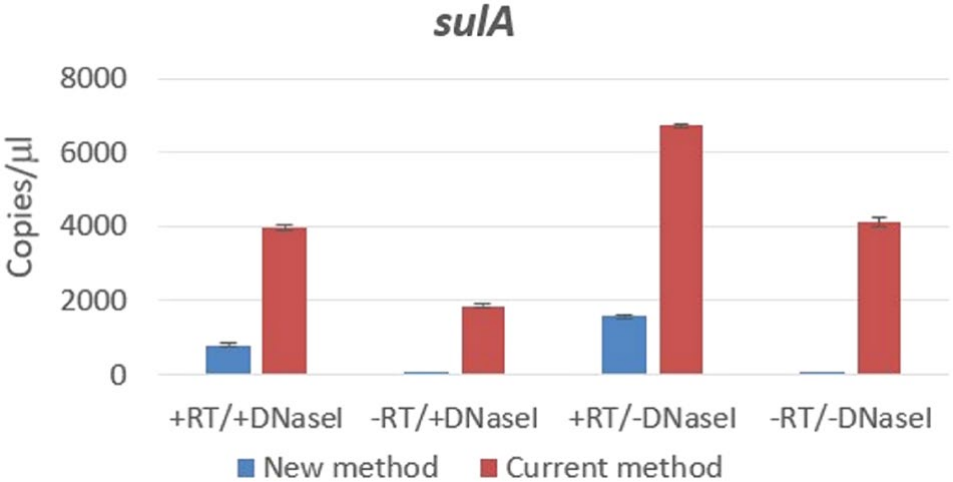
Transcription of *sulA* gene in exponentially growing *E. coli* cells obtained by dPCR using current and new method. Columns represent number of copies/µl and the plotted error bar shows whether or not the events differ with 95% Poisson confidence interval.

**Figure 5:**
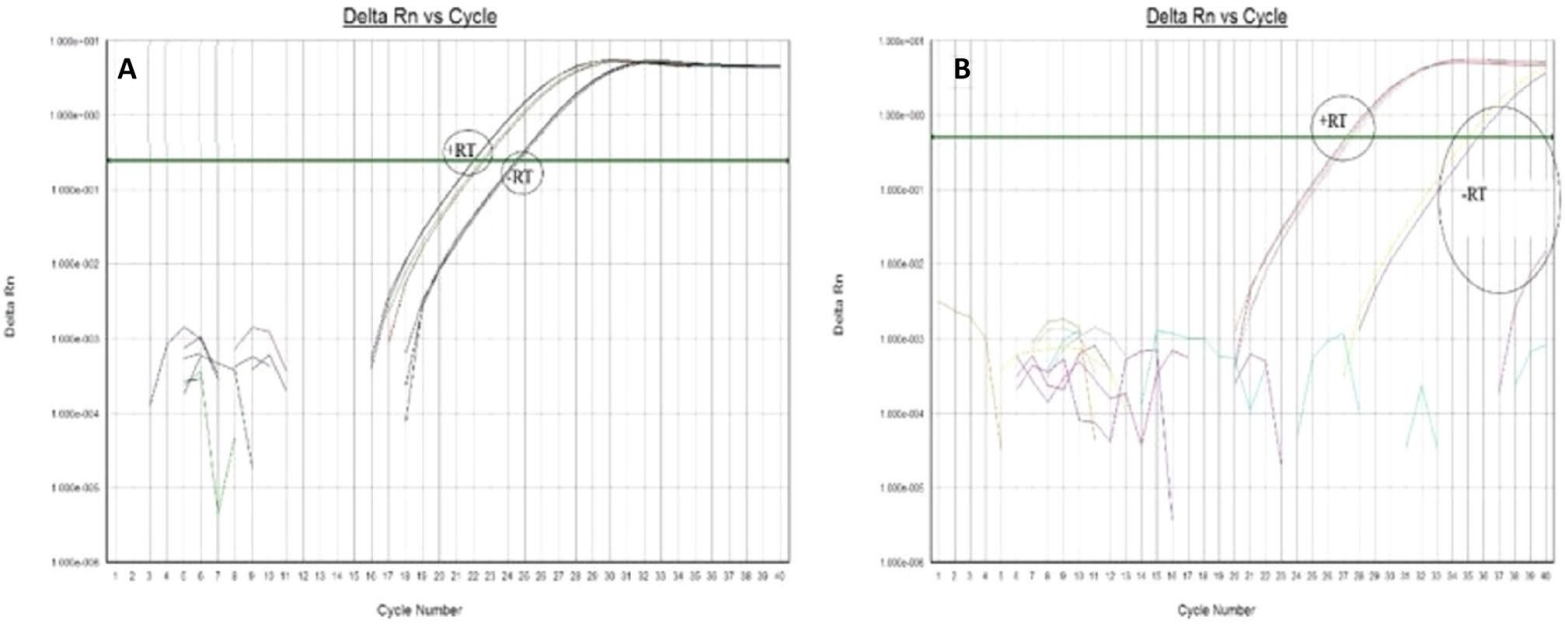
Delta Rn vs Cycle plot of alpha satellite DNA isolated from HeLa cells obtained by qPCR using current method (**A**) and new method (**B**). + RT and – RT represent positive and negative controls, with and without reverse transcription, respectively.

**Figure 6:**
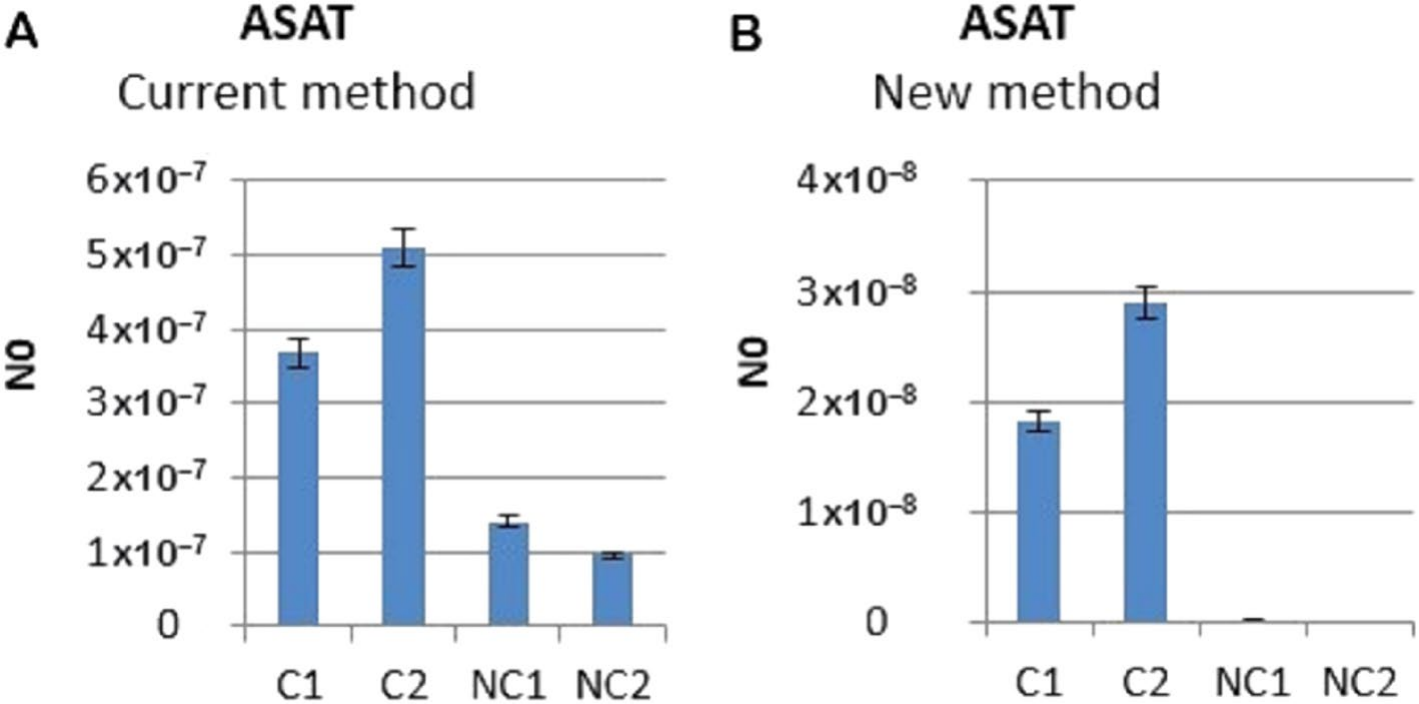
Transcription level of alpha satellite DNA obtained by qPCR using current method (**A**) and new method (**B**). Columns show average of 2 different loaded samples in qPCR experiments performed in triplicate. N0 represents normalized average N0 value for alpha satellite. C represent alpha samples with reverse transcription and NC represents negative controls without reverse transcription and M is 100 bp size marker.

Our proposed new method can be utilized in various experimental investigations and for the purposes of this paper, it has been tested by analyzing three bacterial *E. coli* genes: *ssb*, *sulA* and *recA* (Figs. 2, 3 and 4), and two satellite DNA transcripts: human alpha-satellite (ASAT) (Figs. 5 and 6, and Suppl. Fig. 2) and TCAST1 satellite from *Tribolium castaneum* (Suppl. Fig. 1).

### Analysis of bacterial gene expression

Bacterial genes are a good experimental model to test our method because they do not contain introns in their coding region, removing the possibility of discriminating between transcripts and the DNA according to their different sizes. Hence, the technique could be applied to test the expression of all genes organized with a short or null intron (e.g. viral genes).

The bacterial strain used in this test was transformed with multicopy plasmid carrying a cloned *ssb* gene^9^, which could compete for amplification with *ssb*-cDNA during the transcripts’ quantification by PCR, unless additional DNase I treatments were implemented. The results indicated in Fig. 2 show a large difference (more than 40-fold) in *ssb* transcription levels measured by our method, as compared to the currently used method. This really high level of amplified *ssb* sequence in the latter approach, when reverse transcription was not carried out, and the DNA was eliminated in both RNA isolation and RT steps (Fig. 2A), is likely due to low efficiency of elimination of covalently closed circular plasmid DNA, meaning that it is false (i.e. it does not accurately represent the process of transcription) and is actually caused by DNA contamination.

This is likely a reason for all the observed cases of high levels of *ssb* sequence amplification using classical primers (Fig. 2A). In contrast, *ssb* sequence was amplified by our new method only in those cases when reverse transcription was performed, i.e. when cDNA was created (Fig. 2B). The level of *ssb* sequence amplification did not depend on DNA elimination (Fig. 2B), thus confirming insensibility of our method to the presence of genomic (and plasmid) DNA. Next, we quantified expression of *recA* and *sulA* genes, which are present as single copies in the *E. coli* genome. In accord with the previous assay, no *recA* sequence amplification was observed using our method unless cDNA was created by reverse transcription (Fig. 3). The level of *recA* sequence amplification was, again, independent from genomic DNA elimination from the sample (Fig. 3). Conversely, the current method, which uses standard primers, showed a false positive signal even when reverse transcription step was skipped and the genomic DNA was (obviously incompletely) eliminated by DNase I treatment (Fig. 3).

Finally, analysis of *sulA* gene expression using a modified primer was in accord with the previous assays since amplification of *sulA* sequence occurred only after reverse transcription, i.e. it was specific for cDNA (Fig. 4). Accordingly, no effect was observed after genomic DNA elimination (Fig. 4). In contrast, amplification of *sulA* sequence using standard primers was very different, and was not abolished even in situations where genomic DNA was eliminated and reverse transcription was not performed (Fig. 4); theoretically, the – RT/ + DNase I sample should not contain any cDNA or genomic DNA.

The presented results clearly demonstrate that our method of using a modified primer during cDNA synthesis produces a cDNA-specific PCR signal, which is independent of genomic DNA, and therefore much more accurately quantifies gene expression when compared to the standard, commonly used method, which, unfortunately, does not produce real negative control since there is always possibility to have contaminating DNA in the sample.

### Analysis of satellite DNA expression

Satellite DNA represents one of the best target candidates for demonstrating the effectiveness of our methodology since it is a highly repetitive non-coding genomic DNA, ever-present in large quantities in the sample and therefore difficult, if not impossible, to remove during RNA purification.

Alpha satellite DNA is the most abundant human satellite DNA of 171 bp long, comprising up to 10% of the genome^14^. Figure 5A, shows qPCR results obtained by following the current standard protocol (old method) which implies the elimination of DNA both during the RNA purification and reverse transcription phase. In spite of that, alpha satellite DNA continues to persist in the negative control samples (– RT). Furthermore, since it is not organized into exons and introns, satellite DNA cannot be discriminated from satellite cDNA based on its length; therefore, even a slightest trace of DNA contamination often produces false-positive results. The new method, however, successfully demonstrated the disappearance of the alpha satellite DNA contamination from the qPCR amplification results (Fig. 5B, – RT), as it can be clearly seen also by loading the amplicons on agarose gel (Suppl. Fig. 2): ASAT amplicon of 126 bp long is present only in + RT samples (C: controls) respect to – RT samples (NC: negative control). The same results could be represented as in Fig. 6A (current method) and Fig. 6B (new method), where “N0 value” is the starting concentration of amplicon in the sample and columns show average of 2 different loaded samples in qPCR experiments performed in triplicate (see “Materials & method” section).

The highly abundant satellite DNA TCAST1 has previously been characterized as the major satellite that makes up to 30% of the beetle *Tribolium castaneum* genome, organizing the centromeric as well as pericentromeric regions of all 20 chromosomes^10,13^. Again, using the new method only cDNA was amplified (+ RT samples) and almost nothing of genomic DNA contamination was detected in –RT samples (Suppl. Fig. 1). The results clearly show they are exactly the same as those obtained for human alpha satellite DNA.

## Conclusion

In conclusion, we can affirm that the results achieved through application of our new method of quantifying different types of transcripts are certainly more precise, reproducible and affordable than those obtained by currently used protocols. This is because our method is insensitive to DNA contamination (which usually gives rise to false positive signals) and therefore there is no need for prior elimination of the template DNA. Moreover, skipping the DNA elimination step effectively preserves the RNA from degradation. In that way the two major sources of inherent inaccuracy in transcriptome analyses are avoided.

## Data avalaibility

All data generated or analysed during this study are included in this published article (and its Supplementary Information files).

## Supplementary Information

Supplementary Figures.
