# Supplementary material for: Reverse transcription-quantitative PCR (RT-qPCR) without the need for prior removal of DNA
Source: Sci Rep. 2023 Jul 15;13:11470. doi: 10.1038/s41598-023-38383-4 (PMC10349872; doi:10.1038/s41598-023-38383-4)
Supplement: Supplementary file 1 — Supplementary Figures. [file 41598_2023_38383_MOESM1_ESM.pdf]

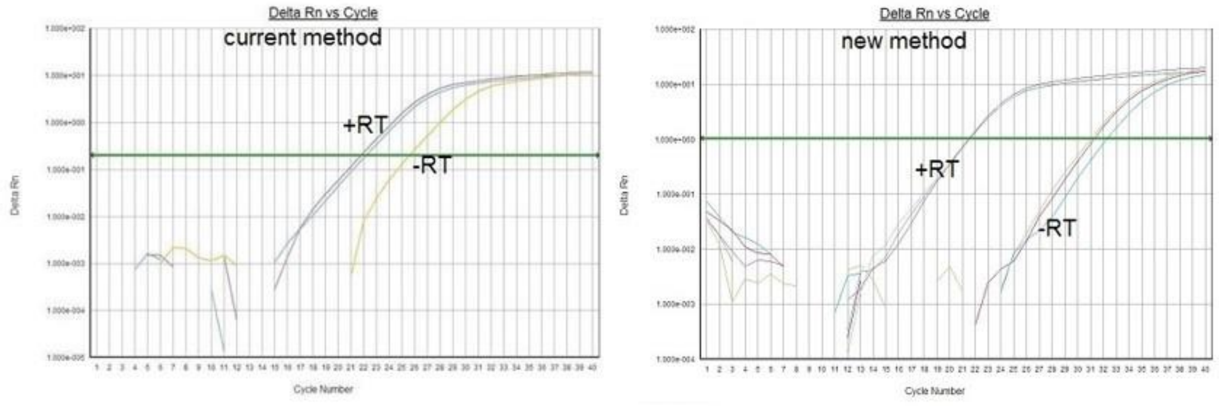

Supplementary Figure 1. Transcription of TCAST1 satellite DNA. Delta Rn vs Cycle obtained by quantitative real-time PCR using current method and new method. +RT and –RT represent positive and negative controls, with and without reverse transcription, respectively.

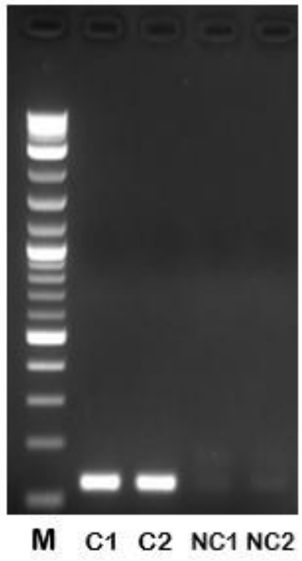

Supplementary Figure 2. Agarose gel with alpha satellite DNA amplicons of 126 bp. NC represents negative controls without reverse transcription; C represents alpha satellite samples with reverse transcription and M is molecular-weight size marker (100 bp – 10 kb).

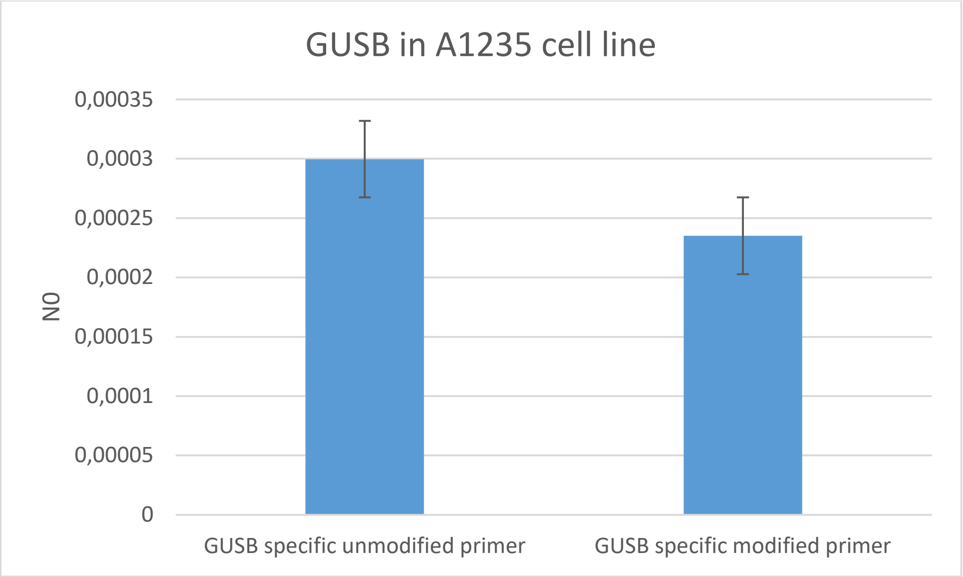

Supplementary Figure 3. Real time PCR analysis of human GUSB gene expression in order to compare the efficiency in reverse transcription using two different specific primers: the unmodified and the modified one by inserted 4 mismatched bases. Columns show average of 2 different loaded samples in qPCR experiments performed in triplicate.
